# Low *VHL* mRNA Expression is Associated with More Aggressive Tumor Features of Papillary Thyroid Carcinoma

**DOI:** 10.1371/journal.pone.0114511

**Published:** 2014-12-09

**Authors:** Boban Stanojevic, Vladimir Saenko, Lidija Todorovic, Nina Petrovic, Dragan Nikolic, Vladan Zivaljevic, Ivan Paunovic, Masahiro Nakashima, Shunichi Yamashita, Radan Dzodic

**Affiliations:** 1 Atomic Bomb Disease Institute, Nagasaki University, Nagasaki, Japan; 2 Laboratory for Radiobiology and Molecular Genetics, “Vinca” Institute of Nuclear Sciences, University of Belgrade, Belgrade, Serbia; 3 School of Medicine, University of Belgrade, Belgrade, Serbia; 4 Center for Endocrine Surgery, Clinic for Endocrinology, Clinical Center of Serbia, Belgrade, Serbia; 5 Department of Surgical Oncology, Institute of Oncology and Radiology of Serbia, Belgrade, Serbia; CCR, National Cancer Institute, NIH, United States of America

## Abstract

Alterations of the von Hippel–Lindau (*VHL*) tumor suppressor gene can cause different hereditary tumors associated with VHL syndrome, but the potential role of the *VHL* gene in papillary thyroid carcinoma (PTC) has not been characterized. This study set out to investigate the relationship of *VHL* expression level with clinicopathological features of PTC in an ethnically and geographically homogenous group of 264 patients from Serbia, for the first time. Multivariate logistic regression analysis showed a strong correlation between low level of *VHL* expression and advanced clinical stage (OR = 5.78, 95% CI 3.17–10.53, *P*<0.0001), classical papillary morphology of the tumor (OR = 2.92, 95% CI 1.33–6.44, *P = *0.008) and multifocality (OR = 1.96, 95% CI 1.06–3.62, *P = *0.031). In disease-free survival analysis, low *VHL* expression had marginal significance (*P* = 0.0502 by the log-rank test) but did not appear to be an independent predictor of the risk for chance of faster recurrence in a proportion hazards model. No somatic mutations or evidence of *VHL* downregulation via promoter hypermethylation in PTC were found. The results indicate that the decrease of *VHL* expression associates with tumor progression but the mechanism of downregulation remains to be elucidated.

## Introduction

Thyroid cancer is the most prevalent type of endocrine malignancy. During the past decades, its incidence has been increasing in many countries [1]. Papillary thyroid carcinoma (PTC) accounts for more than 80% of all thyroid cancers and for 95% of this increase [2]. An important unanswered question relates to the mechanism of this rapid increase, whether it is related to improved detection, or whether a change in the basic nature of thyroid cancer has occurred [3].

PTC is associated with constitutive activation of the RET-RAS-RAF-MAPK pathway, which transduces potent mitogenic and cell survival signals [4], [5]. Pathway activation is usually caused by *RET/PTC* gene rearrangements or activating point mutations in the *BRAF* or *RAS-*family genes. These molecular alterations occur cumulatively in up to 70% of all PTCs [6], [7]. They are nearly mutually exclusive since activation of any single proto-oncogene confers uncontrolled functioning of downstream effectors. In our previous study these genetic alterations were detected in 150 of 266 Serbian PTC patients (56.4%). *BRAF^V600E^* was the most prevalent (84/266, 31.6%), *RET/PTC* rearrangements occurred in 55/266 (20.7%) cases, the *RAS* mutations were the least frequent (11/266, 4.1%) [8].

While activating mutations of *BRAF*, *RAS* genes and *RET/PTC* gene rearrangements promote PTC, other genetic and epigenetic modifications that contribute to malignant progression of this type of thyroid cancer are insufficiently defined. The understanding of these molecular alterations and of their mechanisms may result in the development of novel molecular prognostic and therapeutic strategies for inhibiting oncogenic activity of signaling pathways [9]–[16].

The *VHL* (Von Hippel-Lindau) gene, located on chromosome 3p25, is strongly associated with the development of a dominantly inherited cancer syndrome predisposing to a variety of neoplasms. Von Hippel-Lindau disease is characterized by the development of multifocal, highly vascularized tumors in mesenchymal and neural crest-derived tissues of several organ systems. Clinically most important are tumors of the central nervous system (haemangioblastoma – HB CNS), eye (retinal haemangioblastoma – RB), kidney (renal clear cell carcinoma – RCC), adrenal medulla (pheochromocytoma – PHE), inner ear (endolymphatic sac tumor), and endocrine system (islet cell tumor) [17], [18].

In most VHL patients, autosomal inherited germline mutations can be identified in the *VHL* tumor suppressor gene. To date, more than 1,000 germline and somatic mutations have been reported [19]. Databases of *VHL* gene mutations (www.vhl.org/research/beroud.htm, http://www.umd.be:2020) help to establish genotype–phenotype correlations that allow classification into distinct VHL disease subtypes. Among the characterized gene alterations, point mutations account for about 60%, partial deletions for approximately 30%, and deletions of the entire gene for about 10%. Exceptions to the rule appear to be epigenetic gene silencing and genetic mosaicism [20]–[24].

VHL protein is a part of multiprotein complex with E3 ubiquitin ligase activity which leads to polyubiquitination and proteosomal degradation of specific target proteins. The most extensively studied target of this complex is hypoxia-inducible factor-α (HIF-α), a transcription factor that plays a central role in the regulation of gene expression by oxygen. Under normoxia conditions, the complex marks HIF-α for degradation. In cells that are exposed to hypoxia or lack functional VHL, HIF-α subunits accumulate and bind to HIF-β, forming heterodimers which transcriptionally activate a number of genes whose products are involved in cell adaptation to hypoxia and regulation of angiogenesis, which is one of the key processes in tumorigenesis [25], [26]. Several lines of evidence suggest that the function of VHL is likely to extend beyond its crucial role in oxygen signal transduction, and the loss of its function may result in deregulation of several signalling pathways that have key roles in biological processes such as cell proliferation, cell survival, cell invasion and metastasis [27], [28]. Aberrant expression of *VHL* tumor suppressor gene has been reported in a number of human malignancies, including kidney, colon, breast, gastric cancer and MEN2-associated medullary thyroid cancer [29]–[31].

Arguments that prompted us to study the possible involvement of the *VHL* gene in PTC are: (*i*) *VHL* gene is expressed, and VHL protein is detectable immunohistochemically in thyroid follicular epithelial cells and endothelial cells [32], [33], (*ii*) the expression of VHL protein in nonneoplastic and neoplastic thyroid lesions correlates with tumor differentiation [33], [34], (*iii*) clinicopathological correlations of *VHL* with PTC remain largely unknown.

Therefore, we aimed this study at evaluation of the association between *VHL* status (*VHL* expression, mutations and promoter methylation), and a variety of demographic and cancer characteristics in a group of 264 Serbian patients admitted to our reference center for PTC from 1992 to 2008 [8]. Our work is the first large-scale study of this kind so far.

## Materials and Methods

### Patients, clinicopathological characteristics and detected genetic alterations

A total of 264 patients diagnosed and treated for PTC in the Institute of Oncology and Radiology of Serbia, Belgrade, between June, 1992 and December, 2008 were enrolled. None of the patients had a history of radiation exposure. Pathological diagnosis was based on the WHO standards [35] and confirmed independently by two experienced pathologists (Z.M. and M.N.). Pathological information was retrieved from patients' records. Demographic and tumor characteristics are shown in Table 1.

**Table 1:** Baseline, cancer and treatment characteristics.

Baseline factors	
Age at diagnosis, mean ± SD (range), years	47.6±16.1 (8–82)
Sex	
Male	66 (25.0%)
Female	198 (75.0%)
Sex ratio	0.33
**Cancer characteristics**	
pT category 1	
1	112 (42.4%)
2	41 (15.5%)
3	77 (29.2%)
4	19 (7.2%)
Nodal disease ^2^	175 (66.3%)
Distant metastasis ^3^	9 (3.4%)
Tumor size, mean ± SD (range), mm ^4^	23.4±18.7 (1.5–190)
≤10 mm	60 (22.7%)
>10 and ≤20 mm	86 (32.6%)
>20 mm	107 (40.5%)
Extrathyroidal extension	80 (30.3%)
Vascular invasion	54 (20.5%)
Tumor multifocality	88 (33.3%)
Tumor capsule	107 (40.5%)
Clinical stage	
l	142(53.8%)
ll	10 (3.8%)
llI	32 (12.1%)
IV	80 (30.3%)
Histopathological variant ^5^	
Classical papillary	214 (81.1%)
Follicular	30 (11.4%)
Other	19 (7.2%)
Follow-up period mean ± SD (range), months ^6^	53.1±41.6 (7–187)
Recurrence ^7^	20 (7.6%)
**Treatment modalities**	
Extent of thyroid resection	
Total thyroidectomy	253 (95.8%)
Less than total	11 (4.2%)
Central neck dissection (level VI)	192 (72.7%)
Central + lateral neck dissection (levels VI, II–V)	64 (24.2%)
Radioiodine ablation ^8^	28 (10.6%)

1 T category was not available in 15 cases. ^2^N category was not available in 20 cases. ^3^M category was not available in 5 cases. ^4^Tumor size was not available in 11 cases. ^5^Histopathological variant was not available in 1 case. ^6^Follow-up data were available for 159 patients. ^7^15 patients (75%) have shown recurrence to the regional or local lymph nodes, and 5 (25%) have demonstrated recurrence to distant organs such as the lung, bone, and brain. ^8^Radioiodine ablation was done only in 10.6% of patients due to limited availability of this treatment modality in the country at that time.

Point mutations in *BRAF* exon 15, codons 12, 13, 31, 60 and 61 of *K-, H-* and *N-RAS*, and the *RET/PTC1* and *RET/PTC3* rearrangements were studied in all 264 paraffin-embedded tumor tissues [8].

The protocols of the study were approved by the Ethical Committees of the Institute of Oncology and Radiology and of Nagasaki University. All participants provided their written informed consent to participate in this study.

### Nucleic acid extraction

Tumor tissues were manually microdissected from formalin-fixed paraffin embedded tissue sections obtained from the files of the Department of Pathology, Institute of Oncology and Radiology of Serbia.

DNA was extracted from four 10-µm sections using the Puregene Genomic DNA purification kit (Gentra Systems, Qiagen, Minneapolis, MN, USA). Total RNA was extracted from three 10-µm sections using Recover All Total Nucleic Acid Isolation Kit optimized for FFPE samples (Ambion, Applied Biosystems, Foster City, CA, USA) according to the manufacturer's protocols. DNA and RNA were quantified with a Nanodrop 1000 spectrophotometer (Thermo Fisher Scientific, USA).

### Real-time quantitative PCR

mRNA expression was examined by an optimized two-step real-time quantitative PCR assay [36]. cDNA was synthesized using the iScript cDNA synthesis kit (Bio-Rad, Hercules, CA, USA). The primers were designed with PrimerExpress 2.0 software (Applied Biosystems, Foster City, CA) and are available in the public RTPrimerDB database (http://medgen.ugent.be/rtprimerdb/), gene RTPrimerDB-ID: VHL [37]. PCR amplification mixtures (15 µl) contained SYBR Green PCR Master Mix (12.5 µl, 2x, Applied Biosystems, #4309155), 250 nM of each forward and reverse primer and template cDNA (20 ng total RNA equivalent). Reactions were run on an ABI PRISM 5700 Sequence Detector (Applied Biosystems). The cycling conditions were as follows: 10 min at 95°C, 40 cycles at 95°C for 15 sec and 60°C for 60 sec. All assays were performed in duplicate. After PCR amplification, a melting curve was generated for every PCR product to ensure the specificity of the reaction. Data were analyzed according to the relative standard curve method, in which the transcription levels were normalized by the stably expressed reference gene *GAPDH* (glyceraldehyde-3-phosphate dehydrogenase).

### Sequencing analysis

To screen the *VHL* gene for mutations, we performed direct sequencing of the coding region. The 3 *VHL* exons and their immediately flanking sequences were amplified by PCR as described [38], sequenced in both directions using BigDye Terminator v3.1 Cycle Sequencing Kit and analyzed in an ABI 3730 DNA Analyzer (Applied Biosystems, Foster City, CA, USA).

### Methylation-specific PCR

Methylation-specific PCR (MSP) was done according to Herman et al [39]. SssI methylase (New England Biolabs, Beverly, MA) treated and untreated normal human genomic DNA were used as a positive and negative control, respectively, after bisulphite modification.

### Statistical analysis

All cases in the study were dichotomized according to the *VHL* expression into those (*i*) whose level was below or (*ii*) equal to or greater than median. The subgroups thus defined were compared for baseline factors, clinical and tumor-related characteristics by Fisher's exact test or its extension (http://in-silico.net/tools/statistics/fisher_exact_test) for categorical data or Mann-Whitney test for continuous variables in univariate analysis.

For multivariate analyses of *VHL* expression associations (logistic regression) and of disease-free survival (Cox proportional hazards model), the following variables were tested: age (continuous, years), sex (categorical, M or F), tumor size (continuous, mm), pT category (3+4 *vs*. 1+2, categorical) nodal disease (categorical, yes or no), distant metastasis (categorical, yes or no), the presence of tumor capsule (categorical, yes or no), tumor growth pattern (categorical, papillary or other), multifocality (categorical, yes or no), extrathyroidal extension (categorical: yes or no), vascular invasion (categorical: yes or no), clinical stage (categorical: III+IV *vs*. I+II), mutational status (categorical, including unknown mutation, *BRAF*, *RAS*, *RET/PTC1* and *RET/PTC3*). Non-automatic backward elimination was applied to the full models that included all the variables listed above. Once the most appropriate model was determined, the maximum likelihood estimates of the respective parameters and their 95% confidence intervals were calculated.

Calculations were performed using SPSS 17.0 statistical software package (SPSS, Chicago, IL, USA). The *P*-value less than 0.05 was regarded as indicating statistical significance in all tests.

## Results

### Relationship between VHL mRNA expression and clinicopathological parameters

*VHL* expression was determined in our cohort of PTC tumor samples (n = 264) by real-time quantitative PCR. Patients were subdivided into 2 groups as having high or low *VHL* mRNA expression based on the median of normalized expression values.

As shown in Table 2, the univariate analysis demonstrated that low *VHL* mRNA expression levels are associated with the older age of patients (*P*<0.0001), higher pT category (*P* = 0.002), distant metastasis (*P* = 0.001), advanced clinical stage (*P*<0.0001) and classical papillary growth pattern (*P* = 0.040).

**Table 2:** Expression of *VHL* in thyroid cancers and its association with clinicopathological parameters.

		VHL expression (no. of cases)		
Clinicopathological parameters	Total no. of cases	Low (n = 132)	High (n = 132)	P-value
Gender				
Male	66	36 (54.5%)	30 (45.5%)	0.477
Female	198	96 (48.5%)	102 (51.5%)	
Age				
<45 yr	113	38 (28.8%)	75 (56.8%)	
≥45 yr	147	91 (68.9%)	56 (42.4%)	<0.0001*
N/A	4	3 (2.3%)	1 (0.8%)	
pT category				
1+2	153	64 (41.8%)	89 (58.2%)	
3+4	96	56 (57.3%)	40 (42.7%)	0.002*
N/A	15	12 (2.3%)	3 (0.8%)	
Nodal disease				
N0	69	27 (20.5%)	42 (31.8%)	
N1	175	95 (72.0%)	80 (60.7%)	0.099
N/A	20	10 (7.5%)	10 (7.5%)	
Distant metastasis				
M0	250	119 (90.2%)	131 (99.2%)	
M1	9	8 (6.1%)	1 (0.8%)	0.001*
N/A	5	5 (3.9%)	0 (0%)	
Tumor size				
≤10 mm	60	29 (22.0%)	31 (23.5%)	
>10 and ≤20 mm	86	36 (27.3%)	50 (37.9%)	0.056
>20 mm	107	58 (43.9%)	49 (37.1%)	
N/A	11	9 (6.8%)	2 (1.5%)	
Extrathyroidal extension				
y	80	47 (35.6%)	33 (25.0%)	0.081
n	184	85 (64.4%)	99 (75.0%)	
Tumor multifocality				
y	88	49 (37.1%)	39 (29.5%)	0.240
n	176	83 (62.9%)	93 (70.5%)	
Clinical stage				
I+II	152	50 (37.9%)	102 (77.3%)	<0.0001*
III+IV	112	82 (62.1%)	30 (22.7%)	
Histopathological variant				
Classical papillary	215	114 (86.4%)	100 (75.6%)	
Other	49	18 (13.6%)	31 (23.6%)	0.040*
N/A	1	0 (0%)	1 (0.8%)	
Mutation				
BRAF	85	38 (28.8%)	47 (35.6%)	
RAS	11	5 (3.8%)	6 (4.5%)	
RET/PTC 1	44	22 (16.7%)	22 (16.7%)	0.298
RET/PTC 3	13	10 (7.5%)	3 (2.3%)	
Unknown	111	57 (43.2%)	54 (40.9%)	

*P values less than 0.05 were considered significant, according to the Fisher's exact test for categorical data or Mann-Whitney test for continuous variables.

All other clinicopathological features, including sex, tumor size, vascular invasion, tumor focality, the presence of tumor capsule, and mutational change showed no association with low *VHL* levels.

To further address the correlation between low *VHL* mRNA expression and clinicopathological features, a multivariate logistic regression analysis was performed. Three parameters, i.e. classical papillary morphology (OR = 2.92, 95% CI 1.33–6.44, *P = *0.008), multifocality (OR = 1.96, 95% CI 1.06–3.62, *P = *0.031) and the advanced clinical stage (OR = 5.78, 95% CI 3.17–10.53, *P*<0.0001), but not any other tested, were independently associated with low *VHL* mRNA expression (Table 3). Similar results were obtained when only classical papillary thyroid carcinoma (CPTC) and follicular variant of papillary thyroid carcinoma (FVPTC) were analyzed (S1 Table).

**Table 3:** Multivariate analysis of the correlation between *VHL* mRNA expression and demographic and clinicopathological characteristics of PTC.

Factor	Comparison	Odds Ratio	95% Confidence Interval	P-value
Histological variant	Other 1 vs. Classic	2.92	1.33–6.44	0.008
Multifocality	Present vs. absent	1.96	1.06–3.62	0.031
Clinical stage	III+IV vs. I+II	5.78	3.17–10.53	<0.0001

1 All variants of PTC other than classic papillary combined.

**Figure 1:**
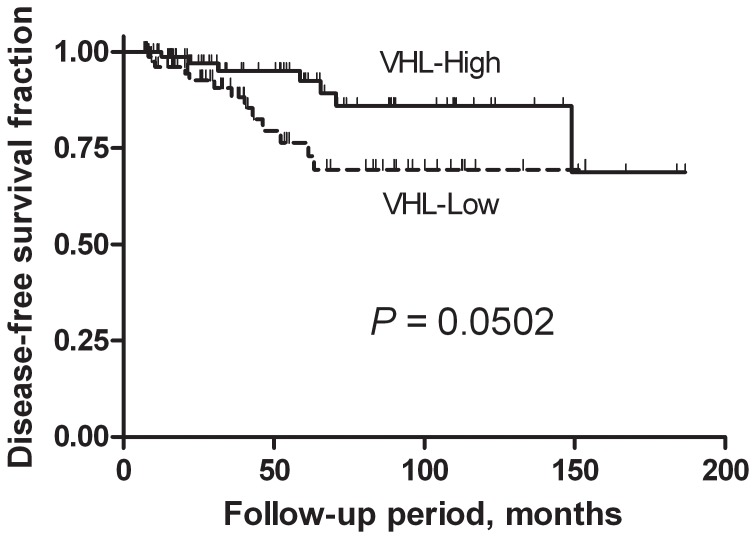
Disease-free survival of PTC patients with low *VHL* expression. Patients with low *VHL* expression had marginal significance (*P* = 0.0502 by the log-rank test) but did not appear to be an independent predictor of the risk for chance of faster recurrence in a proportion hazards model (*P*>0.9).

In disease-free survival analysis, low *VHL* expression had marginal significance (*P* = 0.0502 by the log-rank test, Fig. 1) but did not appear to be an independent predictor of the risk for chance of faster recurrence in a proportion hazards model (*P*>0.9).

### Mutation analysis and methylation status of the VHL gene in PTC

No genetic alterations of the *VHL* gene were found in our tumor series. Mutation analysis was carried out by direct sequencing of the coding region of the *VHL* gene in 264 PTCs. Subsequently, we examined promoter hypermethylation in a group of 130 samples with low mRNA *VHL* expression. None of these samples showed a positive signal with primers specific for methylated DNA. Positive signal was obtained only with primers specific for unmethylated DNA thus providing no evidence of *VHL* gene silencing through methylation.

## Discussion

Various tumor suppressor genes, oncogenes, and intricate networks of signaling cascades have been investigated previously in thyroid tumors [40].

VHL protein is widely expressed in human tissues and its best documented tumor suppressor function is the negative regulation of hypoxia-inducible target genes involved in angiogenesis, erythropoiesis and energy metabolism. Accumulating evidence suggests that VHL may also have HIF-independent and tissue-specific tumor suppressor functions since it has been implicated in diverse cellular processes, including regulation of the extracellular matrix (ECM) and cell invasion [41]–[44], cytoskeletal stability [45] and cell-cycle control and differentiation [46]–[48]. Several studies suggest that VHL plays a critical role in regulating apoptotic pathways in renal cell carcinoma [49]–[51]. According to a recent report, VHL may be a positive regulator of TP53, providing insight into another potential mechanism by which VHL loss of function may contribute to carcinogenesis [52]. The role of VHL in thyroid cancer development is obscure. Since it has been reported that normal follicular epithelium shows a strong expression of VHL protein and that a differential expression of VHL protein in nonneoplastic and neoplastic thyroid lesions is in proportion to the level of tumor differentiation [32], [33], [34], it is reasonable to assume that VHL may be involved in the development of the most common type of thyroid cancer, PTC.

These reports prompted us to investigate the possible role of *VHL* as a classic tumor suppressor gene and a potential association of its expression level with the development and clinicopathological features of PTC.

On univariate analysis, low *VHL* expression, beside the older age of patients (*P*<0.0001), higher pT category (*P* = 0.002), distant metastasis (*P* = 0.001), was also strongly associated with the more advanced clinical stage (*P<*0.0001, Table 2). No significant correlations were detected between *VHL* expression and any other clinicopathological parameters. On multivariate analysis, low *VHL* expression was associated with the advanced clinical stage (OR = 5.78, 95% CI 3.17–10.53, *P*<0.0001). This result confirms that low *VHL* expression level is in correlation with more advanced disease and supports its potential usefulness in identifying patients at risk for disease progression.

Multivariate analysis also showed that low *VHL* expression was independently associated with classical papillary growth pattern (OR = 2.92, 95% CI 1.33–6.44, *P = *0.008) and tumor multifocality (OR = 1.96, 95% CI 1.06–3.62, *P = *0.031). Based on the results presented in Table 2 and Table 3, *VHL* expression may be expected to be lower in PTC with classical papillary growth pattern as compared to follicular variant and other histological variants of PTC. It therefore would be interesting to compare *VHL* expression in PTC with follicular adenoma (FA) and follicular thyroid carcinoma (FTC) in further investigations. PTCs frequently occur as multifocal or bilateral tumors [53], [54]. Several findings suggest that the multiple foci in multifocal PTC represent intraglandular spread from a single primary tumor [54], [55] and tumors of this origin are likely to be aggressive and accordingly, require more extensive treatment [56]–[58]. Our data suggest that *VHL* depression favors the selection of more aggressive cancer cells, which generate multifocal tumors. Our IHC data supported the loss of VHL in more advanced tumors (S1 Figure).

To the best of our knowledge, this is the first demonstration of the association between *VHL* levels and clinicopathological parameters of PTC. Moreover, our study is the first evidence of involvement of *VHL* in PTC. As for other types of cancer, Zia et al. showed that *VHL* had a low level or was not expressed in highly aggressive breast cancer cell lines and that it affected cell motility and invasiveness. They also found a significantly lower level of *VHL* in higher grade breast cancer tumors compared to those of a lower grade, as well as in tumors from patients with nodal and distant metastasis [59]. According to a recent study of Liu et al., the loss of *VHL* increases ovarian cancer cell aggressiveness [60]. Chen et al., demonstrated that reduced pVHL expression was associated with decreased apoptosis and a higher grade of chondrosarcoma [61]. Hoebeeck et al., in a study on 62 neuroblastoma patients, obtained a strong correlation between the reduced levels of *VHL* and lower probability of patients' survival [62].

Our analysis showed that for disease-free survival, low *VHL* level was marginally significant on univariate analysis (*P = *0.0502, Fig. 1). On multivariate analysis *VHL* expression was not a variable conferring risk for chance of faster recurrence while others (specifically, younger age and advanced pT category) were. From the clinical point of view, our series is characterized by the short to medium follow-up period (32.0; 53.5; 89.6 months, the 25%, 50%, and 75% quartiles, respectively, even though we set the expected duration of follow-up of >6 months as an inclusion criterion for DFS analysis), and it is possible that a longer follow-up is required to evaluate its prognostic significance.

The major mechanisms of *VHL* gene inactivation are intragenic mutations, mitotic recombination events, and hypermethylation of the promoter region. Mutations in the *VHL* gene occur in various inherited tumors associated with VHL disease as well as in some sporadic tumors such as clear-cell renal carcinomas, hemangioblastomas and sporadic pheochromocytoma [21]–[25]. On the other hand, studies examining a variety of other sporadic tumors, including breast, colon, lung, and prostate cancers, have found that somatic *VHL* mutations are rare in histological tumor types that are not observed in VHL disease [29]. This is consistent with the results of our analysis. Although loss of heterozygosity at chromosome 3p was found in 86% of FTCs and 29% of PTCs including the *VHL* gene locus (3p25) [63], no evidence for mutations or homozygous deletions of the *VHL* gene could be found in our tumor series as all *VHL* exons were amplified by polymerase chain reaction in all samples.

*VHL* gene is silenced by methylation in 20–30% of patients with renal cell carcinoma [64], [65] and other tumor types such as multiple myeloma (30%) [66]. Hatzimichael at al., also reported that methylation of the *VHL* promoter is a common event in plasma cell neoplasias and might have clinical utility as a biomarker of bone disease [67]. There are several published reports describing epigenetic modifications in thyroid carcinomas. In a panel of analyzed tumor suppressors, promoter hypermethylation of *CDH1*, *p16INK4A*, *RASSF1A* and *SLC5A8* in malignant thyroid tumors was confirmed [68]–[70]. Only one research paper has analyzed the methylation status of *VHL* in patients with thyroid cancer. In this paper, Migdalska-Sek at al. assessed and compared the methylation level of 8 tumor suppressor genes, *ARHI, CDH1, KCNQ1, MEST, p16INK4A, RASSF1A, SLC5A8* and *VHL* in PTC tumor tissues and matched adjacent noncancerous thyroid tissues [71]. The highest methylation rate, i.e. 100% of methylated specimens, was found in 4 genes: *ARHI*, *CDH1*, *p16INK4A* and *RASSF1A*. The frequency of promoter methylation of the *VHL* gene was the lowest, in both cancerous and noncancerous tissues. Analysis of our PTC samples with reduced *VHL* levels has not found evidence for *VHL* gene silencing through methylation, suggesting that the reason for the decreased *VHL* expression might be posttranscriptional downregulation. Consistent with this hypothesis, Valera et al. showed that microRNAs could act as an alternative mechanism of *VHL* inactivation, which was correlated with tumorigenesis in clear-cell renal carcinoma. They found that tumor samples that expressed increased amount of miR92a showed decreased levels of *VHL* mRNA [72], suggesting the possibilities that miR92a or some other microRNAs may regulate *VHL* gene expression in PTC. However, since only 1 primer pair was used for MSP in our experiments, we cannot rule out the possibility of the presence of methylated CpG islands in the promoter regions that were not covered.

## Conclusions

In summary, our study shows that low *VHL* expression is associated with more aggressive tumor features of PTC and thus opens a new perspective for research into the role of *VHL* inactivation in PTC progression. Since the results revealed the absence of common genetic or epigenetic modifications responsible for *VHL* gene downregulation, further analysis including a more detailed understanding of gene regulation and VHL interactions, is required to elucidate the mechanism of *VHL* effect in thyroid cancer.

## Supporting Information

S1 Figure **Immunohistochemical staining of VHL protein in human thyroid cancer.** Normal epithelial cells (A) stained strongly or moderately for pVHL in the cytoplasm whereas PTC (B) and poorly differentiated thyroid carcinoma (PDTC) (C) showed a lower degree of staining or no staining at all.
(TIF)

S1 Table **Regression model for the follicular variant of papillary thyroid carcinoma vs. classical variant of papillary thyroid carcinoma.**
(DOC)
