# Supplementary figures and images for: Low VHL mRNA Expression is Associated with More Aggressive Tumor Features of Papillary Thyroid Carcinoma
Source: PLoS One. 2014 Dec 9;9(12):e114511. doi: 10.1371/journal.pone.0114511 (PMC4260854; doi:10.1371/journal.pone.0114511)

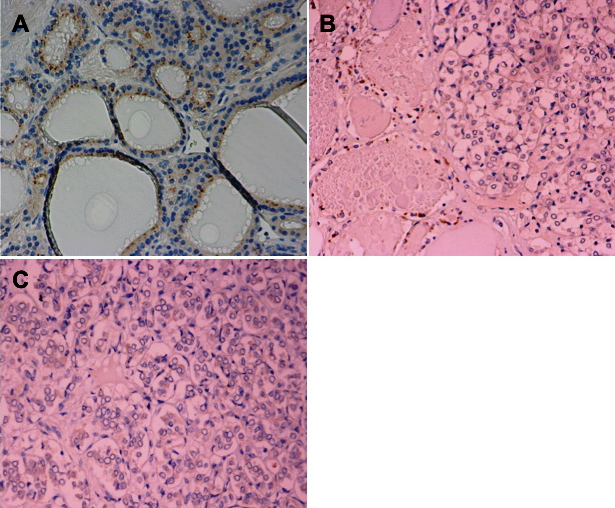

Supplement: S1 Figure — Immunohistochemical staining of VHL protein in human thyroid cancer. Normal epithelial cells (A) stained strongly or moderately for pVHL in the cytoplasm whereas PTC (B) and poorly differentiated thyroid carcinoma (PDTC) (C) showed a lower degree of staining or no staining at all. (TIF) [file pone.0114511.s001.tif]
