# Supplementary material for: Low VHL mRNA Expression is Associated with More Aggressive Tumor Features of Papillary Thyroid Carcinoma
Source: PLoS One. 2014 Dec 9;9(12):e114511. doi: 10.1371/journal.pone.0114511 (PMC4260854; doi:10.1371/journal.pone.0114511)
Supplement: S1 Table — Regression model for the follicular variant of papillary thyroid carcinoma vs. classical variant of papillary thyroid carcinoma. (DOC) [file pone.0114511.s002.doc]

**Table S1. Regression model for the follicular variant of papillary thyroid carcinoma vs. classical variant of papillary thyroid carcinoma.**

| **Factor** | **Comparison** | **Odds Ratio** | **95% Confidence Interval** | **P-value** |
| --- | --- | --- | --- | --- |
| Histological variant | Follic. vs. Classic | 2.62 | 1.01-6.79 | 0.047 |
| Multifocality | Present vs. absent | 1.96 | 1.04-3.69 | 0.037 |
| Clinical stage | III +IV vs. I + II | 5.18 | 2.80-9.62 | < 0.0001 |

^1^All variants of PTC other than classic papillary combined.
